# PD70 Situational Diagnosis Of Health Technology Assessment In Mexico: Challenges And Opportunities For Institutional Strengthening

**DOI:** 10.1017/S026646232510202X

**Published:** 2025-12-29

**Authors:** Verónica Gallegos-Rivero, Karla Torres-Velasco, Sergio Vidal-Flores, Mariana Beatriz Pineda-López

## Abstract

**Introduction:**

Mexico has an institutionalized health technology assessment (HTA) process. However, structural changes in the system and new trends make it necessary to rethink strategies for strengthening and institutionalizing HTA. For this reason, the National Center for Technological Excellence in Health (CENETEC), through the Inter-institutional HTA Working Group, in which the main institutions of the sector participate, coordinates sectoral efforts and has implemented an instrument to collect information to achieve this objective. This study aimed to provide a comprehensive overview of current HTA practices in Mexico to inform future capacity building initiatives within the health system.

**Methods:**

The study used a structured survey to assess the state of HTA across healthcare institutions in Mexico. The survey included 115 participants from all institutions and diverse fields, including medicine, biomedical engineering, economics, and health sciences. The survey questions addressed awareness, implementation, and perceived gaps in HTA. Data collection focused on institutional participation, existing frameworks, and areas requiring improvement. Responses were analyzed to identify trends, challenges, and opportunities for HTA enhancement.

**Results:**

The survey received 115 responses, predominantly from medical professionals, researchers, and economists. Findings revealed widespread awareness of HTA but noted limited institutional implementation. Key challenges included insufficient training, lack of sensitization among decision-makers, and the need for defined expert groups in health institutions. Notable areas requiring focus were cost effectiveness, clinical safety, and broader societal impacts. Participants emphasized the importance of developing consistent HTA methodologies and fostering collaboration across institutions. Despite challenges, the study highlighted significant interest and expertise among stakeholders, providing a solid foundation for advancing HTA practices in Mexico.

**Conclusions:**

The study underscored the importance of strengthening HTA practices in Mexico. While awareness of HTA exists, consistent implementation and capacity building are needed to optimize decision-making. Developing expert groups and enhancing training among stakeholders are crucial steps. These findings offer a roadmap for institutional collaboration and the advancement of equitable, evidence-based HTA frameworks to improve Mexico’s healthcare outcomes.

